# Positive associations between day-level physical activity and healthy eating in participants of a health promotion course: an exploratory study

**DOI:** 10.1007/s10865-026-00642-w

**Published:** 2026-03-07

**Authors:** Matthias Burkard Aulbach, Jens Blechert

**Affiliations:** Department of Psychology, Centre for Cognitive Neuroscience, Paris-Lodron-University of Salzburg, Hellbrunner Strasse 34, 5020 Salzburg, Austria

**Keywords:** Ecological momentary assessment, Physical activity, Eating behavior, Multiple health behavior change, Self-regulation, Lifestyle change

## Abstract

Unhealthy diets and a lack of physical activity (PA) often concur in the same individuals and jointly contribute to overweight and related diseases. Limited self-regulation abilities commonly result in a gap between intentions to eat better and move more and respective behavior enactment (‘intention-behavior gap’, IBG). What is less clear is whether the two health behavioral domains eating and physical activity facilitate or inhibit each other. While cybernetic and resource depletion models would predict that engaging in one behavior leads to *reduced* effort in the other behavior (compensation), motivational accounts predict the opposite: more PA should *increase* healthy eating and vice versa (transfer). Elucidating such relationships across time requires multiple assessments from the same individuals and sufficient incidences of both behaviors, and hence, relatively long assessment periods. This study aimed to investigate cross-behavior relations in participants for whom both behaviors contribute to a higher-order health goal by using data obtained through ecological momentary assessment. 25 participants (20 women, 5 men, mean age = 56) of a health-insurance organized health promotion course provided daily data on intentions, self-efficacy, and behavior enactment for PA and healthy eating for eight weeks, with the average participant submitting data on 42 days. We found that cross-behavior associations on all assessed variables as well as IBGs (computed as the difference between intentions and behavior) were positive. This positive relation between IBGs was independent of day-level variables typically implicated in self-regulation (stress, mood, tiredness, and hours of sleep during the preceding night). Results contradict cybernetic and resource depletion models of self-regulation and speak more to effects of positive transfer between behaviors in this sample. Providing feedback on such positive associations might be a beneficial intervention component to encourage parallel engagement in PA and healthy eating. Future research should aim to further identify other within- and between-person factors (e.g., willpower beliefs) contributing to the PA-healthy eating association.

## Introduction

Unhealthy diets ( i.e., diets high in saturated fats, sugar, and salt and low in nutrients; World Health Organization, 2019), a lack of physical activity (PA; ‘bodily movement produced by the contraction of skeletal muscle that increases energy expenditure above the basal level’; Manley, 1996, p. 20), and resulting overweight as well as metabolic syndrome are major risk factors for the development of type-2 diabetes or cardio-vascular diseases (Hussain et al., 2007; World Health Organization, 2023). A large body of research demonstrates that unhealthy diets and physical inactivity tend to co-occur in the same individuals (Cook et al., 2013; de Vries et al., 2008; Meader et al., 2016), and health risk is particularly high in those displaying multiple health risk behaviors at a time (Spring et al., 2012). Based on this, it has long been recognized that successful behavior change interventions should tackle *both* diet and physical activity and, for example, evaluated diabetes prevention programs have incorporated both types of behavior (Hussain et al., 2007; Uusitupa et al., 2019).

**Fig. 1:**
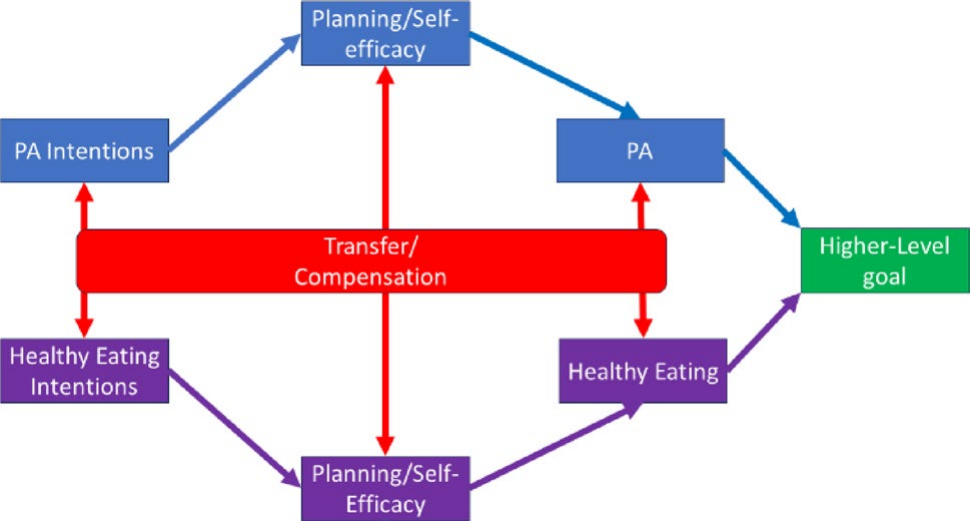
Simplified version of the Compensatory-Carry-Over Action Model (Lippke, 2014). Red arrows indicate cross-behavior relations for different aspects of the self-regulation process (Color figure online)

While behavioral interventions often tackle multiple behaviors at a time (James et al., 2016; Johnson et al., 2014; Mc Sharry et al., 2015; Meader et al., 2017; Nigg & Long, 2012), classical theories of (health) behavior are usually formalized on a ‘per-behavior’ level or generically (that is, applicable to any kind of behavior), both of which implies that specific health behavior models need to be developed and tested ‘one at a time’. This results in a limitation to only one target behavior (e.g., nutrition). A second behavior, for example PA, would have to be conceptualized independently using the same model and similar predictions. This is an obstacle to better understanding dynamics between changes in different behavior domains. To open up the view to multiple behavior (change) the Compensatory-Carry-Over Action Model (CCAM, see Fig. 1; Lippke, 2014) explicitly points to the cross-connections in the self-regulatory process between different types of behaviors (see also Geller et al., 2017). Fundamentally, the CCAM follows classical models like the Health-Action Process Approach (HAPA; Schwarzer et al., 2011) by postulating that people form intentions which, through self-regulatory phenomena like planning and self-efficacy (the belief in being able to overcome barriers), are translated into respective behavior. The CCAM goes beyond the HAPA by recognizing that several different health behaviors often serve a unifying higher-level goal and therefore tap into the same self-regulatory processes. For example, both increasing PA and improving one’s diet serve the higher order goal of preventing the development of type-2 diabetes. From this insight, the model defines several possible mechanisms that can produce effects in one behavioral domain from processes happening in the other behavior’s self-regulation process on the level of intentions, self-efficacy, and behavior (Geller et al., 2017; Grembowski et al., 1993; Lippke et al., 2012).

Predictions about the ways in which two behavioral domains likely influence each other, however, depend on the theoretical account of self-regulation. Motivational theories (Bandura, 2013; Schunk & Usher, 2012; Weiner, 1985) emphasize the role of self-efficacy: achieving goal progress in one behavioral domain strengthens the belief in one’s own capabilities and thus *energizes* efforts in the other domain. Such ‘transfer cognitions’ would thus lead to a positive relation between the two behaviors (‘carry over’). In contrast, cybernetic models of self-regulation postulate that progress towards a goal leads to ‘*coasting*’, that is, a reduction of goal-striving when things are on track towards goal attainment (Carver & Scheier, 2012). When two behaviors serve the same goal, engagement in one behavior should thus lead to reduced effort in the other. This can go along with ‘compensatory health cognitions’ (Knäuper et al., 2004), that is, beliefs that engaging in one health behavior can compensate for (neglecting) another (e.g., doing sports compensates the negative effects of unhealthy snacks regarding overall energy balance). Finally, depletion models that regard self-control as a limited resource (Baumeister, 2000) would predict that effortfully regulating one behavior leaves less energy for self-regulation in another behavioral domain, leading to a negative relation between both behaviors. According to this model, a wide range of factors can reduce self-control resources: being stressed (Tice et al., 2001), tired (Hagger, 2010), or in a bad mood (Tice et al., 2001, 2007) have all been hypothesized and empirically demonstrated to reduce self-control resources, making successful self-control less likely. When examining cross-behavior relations, this would imply that the presence of such depleting factors should explain any cross-behavior association (because they influence both behaviors to the same degree) or lead to a negative relation between them (because resources only suffice for regulating one behavior but not the other).

Research has demonstrated the value of explicitly accounting for cross-connections between multiple behavior types and has shown that individuals who are physically inactive also tend to have poorer diets (Cook et al., 2013) and higher fat intake (Chevance et al., 2020). Those with stronger intentions to change one behavior are also more likely to intend changing the other (Cook et al., 2013). In line with this, an intervention targeting self-regulation in PA also found changes in fruit and vegetable intake (Fleig et al., 2011) but changes in PA did not predict changes in eating behavior in another study (Dutton et al., 2008). A lifestyle weight control program that promoted exercise motivation contributed to more successful self-regulation in eating behavior (Mata et al., 2009) and compensatory and transfer cognitions play a role in this self-regulation process and in the influence of exercise on healthy nutrition (Fleig et al., 2014, 2015). Generally, changing one behavior often leads to the initiation of behavior change in another behavioral domain in weight management interventions (Johnson et al., 2014).

These studies provide good evidence that investigating multiple behaviors is important but are limited by the type of used data. Most studies use data from a single or few timepoints to study associations between predictors and behaviors. This results in findings on a *between-individual* level: individuals with more PA also show better eating (and vice versa). However, theories of change typically make claims about processes *within* individuals and therefore need to collect data and model processes on that level (Johnston & Johnston, 2013). Ecological momentary assessment (EMA) is a method to obtain data from participants (typically) via smartphones in high temporal resolution over extended periods of time (Bolger & Laurenceau, 2013) and such intensive longitudinal data allows examining co-occurrence of different behaviors across time within individuals. Practically speaking, it allows investigating whether – in a given individual – *days* with better diets are also *days* with more physical activity (rather than *individuals* with more PA being *individuals* with healthier diets).

Few studies have used such intensive longitudinal data to investigate the temporal clustering of PA and healthy eating. One study that did so found no temporal covariation between sleep, caloric intake, and physical activity (Hooker et al., 2020). Another study showed that an intervention to promote fruit and vegetable consumption also helped to reduce consumption of non-core foods like candy or cake (but not to increase PA). In addition, those researchers found that those receiving the intervention to eat more fruit and vegetables (but not those in the control group) showed a negative day-to-day relation between physical activity and the consumption of fruit and vegetables, indicating compensation (Nigg et al., 2021). In a similar vein, participants in another study tended to consume more water, fruit, and vegetables (indicating transfer), but also more sugar-sweetened beverages and fried fast food (indicating compensation) when being more physically active than usual (Maher et al., 2020). In another study, physical activity led to more healthy eating in the future and vice versa over the course of the day and such sequences of healthy behaviors were associated with increased well-being. On the flipside, unhealthy eating was associated with later lack of PA and vice versa (Dohle & Hofmann, 2019).

The cited studies provide a good starting point for understanding how PA and eating behavior interact by showing their association over time, but results seem mixed as to whether healthy behaviors tend to be positively or negatively associated over time. One crucial limitation in the literature is the exclusive focus on behavior, omitting aspects of the self-regulation processes involved in the concurrent goal-striving in both behavioral domains. Behavior often falls short of intentions, a widely documented phenomenon termed the ‘intention-behavior gap’ (IBG; Sheeran, 2002; Sheeran & Webb, 2016) which has implications for further goal striving, for example through self-efficacy (Carver & Scheier, 2012; Lippke, 2014; Weiner, 1985). Earlier research using EMA has shown that intentions are often not translated into behavior for both PA (Haag et al., 2023; Maher et al., 2016; Pickering et al., 2016) and eating behavior (Aulbach et al., 2024; Reichenberger et al., 2019) also from day to day. A sole focus on behavior cannot really speak to self-regulation failure unless accounting for the respective intentions. For example, successful longer-term goal pursuit can involve temporarily pausing goal striving and such ‘self-licensing’ differs from unsuccessful intention implementation in its effects on future efforts towards the goal (Prinsen et al., 2018, 2019). To make such a distinction, measuring daily intentions and self-efficacy alongside respective behavioral enactment is crucial. The present study thus explores transfer and compensatory interactions between eating and PA for different variables relevant for the self-regulation process: intention, self-efficacy, and behavior. Assessing both intentions and behavior also allows determining IBGs in either behavioral domain and the cross-behavior association of IBGs over time. To investigate such relations, we use data from a naturalistic study sample and context – specifically, participants were partaking in a health insurance funded secondary prevention course promoting lifestyle change in PA and eating. This self-selected sample thus had a higher-order health goal that requires engaging in both PA and healthy eating and was supported in changing both behaviors during the study period. During the course, they reported eating and PA behaviors alongside self-regulatory cognitions (intentions and self-efficacy) daily (in the evening ) across eight weeks. In addition, participants reported on factors that have been shown to influence self-regulatory success, that is, mood, tiredness, and stress (Hagger, 2010; Tice et al., 2001, 2007) and thus could influence the relation between behaviors.

The aim of the present study is thus to examine day-level relations between core aspects of the self-regulatory process (intention, self-efficacy, behavior, and IBG) for PA and healthy eating within participants with a higher-order health goal that requires engagement in both behaviors.

## Materials and methods

Due to its exploratory nature, this study was not pre-registered. The study was approved by the Ethical Review Board of the University of Salzburg (decision number EK-GZ 11/2020). Data, analysis code, Supplementary Material, and the pre-print of this manuscript can be found at https://osf.io/ubvn5/.

### Participants

Study participants were recruited from individuals enrolled in a health promotion course offered by a local health insurance provider. The course was advertised via a newsletter as a health promotion course for people with a high BMI, or elevated blood sugar or fat levels, who intended to improve their diet and physical activity levels. The only requirement to participate in the course was to be a customer of the health insurance provider. Participation in the study was voluntary and not connected with course participation, so not all participants in the course took part in the study. Criteria for study participation was sufficient command of German language to understand all items and instructions and to not have health issues that prevented engaging in PA. Of the 34 course participants, *n* = 28 individuals signed up for the current study. Since calculating an IBG (see below) from one day to the next requires a pair of subsequent days, we only included individuals who provided at least 10 day-pairs (i.e., data was input for two consecutive days at least 10 times) which led to the exclusion of 3 participants 1. This resulted in a sample of 25 participants (20 women, 5 men; mean age = 56 years (*sd* = 13.98)). The sample size was thus determined by the number of course participants willing to participate in the study and providing sufficient data.

### Course contents

Course content was based on the ‘Pre-dias’ program (Kulzer et al., 2009), an evidence-based type-2-diabetes prevention program implemented in small groups (three groups of 10–15 participants), all of which presented the same content. The intervention consisted of a total of six in-person sessions held by licensed clinical psychologists, each session lasting three hours. After four weekly sessions (the core phase), a break of six weeks followed. Two more weekly “booster sessions” concluded the course after the break. The session contents included educational material about overweight and associated health risks, diet, physical activity, as well as goal setting, self-regulation, habits, barrier management, and other psychological contents relevant to goal pursuit. In addition, participants had one group session with a dietitian and a cooking class and had free access to physical activity classes.

### Procedure

At the beginning of the course, participants gave informed consent and then filled out a range of questionnaires. At the same occasion, researchers helped participants install the EMA application m-path (Mestdagh et al., 2023) on participants’ own mobile devices and gave instructions regarding items in the daily signals. Participants received daily signals (smartphone push notifications prompting input into the app) at 9pm for 56 days, starting with the first course session and ending three weeks after the conclusion of the course’s core phase. Data collection lasted from April to June 2023. The assessment schedule was chosen to capture day-to-day fluctuations and balancing participant burden with amount of collected data points.

### Measures

*Baseline measures* included questionnaires about smoking, alcohol consumption, and general well-being which are not relevant for the current study. Further, a food frequency questionnaire and the international physical activity questionnaire (IPAQ ; Craig et al., 2003) were used to assess general eating patterns and physical activity during the past week, respectively. We further measured implicit theories of willpower (Job et al., 2010) as well as transfer cognitions using the Transfer Cognitions Scale TRACS (Fleig et al., 2014) and compensatory health beliefs adapted to relate to physical activity and eating (Fleig et al., 2015). Since we focused on within-individual associations here due to a lack of between-subjects statistical power, these data are presented in the Supplementary Materials.

**Table 1:** Means, standard deviations, question wording (translated from German by the authors), and scale anchors for all variables

Variable	Mean	SD	Item	Scale and anchors
Mood	73.1	20.7	How was your mood today?	0-100 [very bad – very good]
Hours of sleep	7.24	1.45	How many hours did you sleep last night?	1–15
Tiredness	37.3	24.7	How tired were you during the today?	0-100 [not at all – very]
Stress – time pressure	34.3	25.1	I had the feeling not to have enough time for my tasks today.	0-100 [not at all correct – completely correct]
Stress – burden	36.6	26.6	I feel very burdened today.	0-100 [not at all correct – completely correct]
PA intentions	67.1	23.4	How much are you intending to be physically active for your health tomorrow?	0-100 [not at all – very]
PA self-efficacy	74.0	18.2	How confident are you that you can implement this intention?	0-100 [not at all – very]
PA	56.7	27.0	How physically active were you today for your health?	0-100 [not at all – very much]
Eating intentions	72.0	20.1	How much are you intending to eat healthily tomorrow?	0-100 [not at all – very]
Eating self-efficacy	73.3	20.0	How confident are you that you can implement this intention?	0-100 [not at all – very]
Eating	61.6	23.7	How healthily did you eat today?	0-100 [very unhealthily – very healthily]

*EMA measures.* At the daily prompts, participants were asked to respond to a short set of questions, including their retrospective report of behavior for the day, intentions to engage in the two behaviors the next day, and self-efficacy regarding those intentions, with all items adapted from earlier studies (Haag et al., 2023; Maher et al., 2016). Further, they retrospectively indicated their mood, stress, tiredness, and hours of sleep for that day (see Table 1 for wording of all items). All items were rated on a horizontal slider ranging from 0 to 100 (except hours of sleep) and participants received instructions on how to fill them out from the research team. For the behavior items, participants were instructed to indicate their own perception of their behavior on that day. For PA, the instruction was that 0 indicated no activity at all and 100 indicated the most PA the participant could possibly do in their current condition. An example for giving a score in the low range was if someone had once taken the stairs instead of using the elevator but not moved otherwise for health reasons. Participants were further instructed not to include PA they had to engage in for work but instead focus on PA they had voluntarily performed for health reasons. This was done because work-based PA is not performed for the higher-order health goal and therefore would not contribute to the self-regulatory process under study. For healthy eating, participants received input during the course about elements of a healthy diet, including intake of fruit, vegetables, and fiber, and low intake of saturated fat, salt, and sugar, based on national dietary guidelines (BMSGPK, 2024).

We decided to measure all variables as one broad subjective rating with a 0–100 virtual analogue scale (in line with earlier studies, e.g. Aulbach et al., 2024; Dohle & Hofmann, 2019; Reichenberger et al., 2021; van Alebeek et al., 2024) rather than measures more closely related to actual behavior. This was done because we expected participants to differ widely regarding eating patterns and levels of physical activity and we were mainly interested in their own perceptions of behavior as this perception might be more important for the self-regulatory process than objective behavior (Smyth et al., 2023) because different individuals will regard different behaviors as success or failure: an initially very inactive person might perceive taking a daily walk as an active day whereas for an already active person this would be a rather sedentary day. We assume that this perception of goal progress (rather than the actual behavior) should have the most important downstream effects on other (and future) self-regulatory efforts (Kavanaugh et al., 2015; van Alebeek et al., 2024). Since even small initial goal progress can be important when starting behavior change, we decided to use a low threshold for reporting even small amounts of PA.

### Data preparation and analysis

**Fig. 2:**
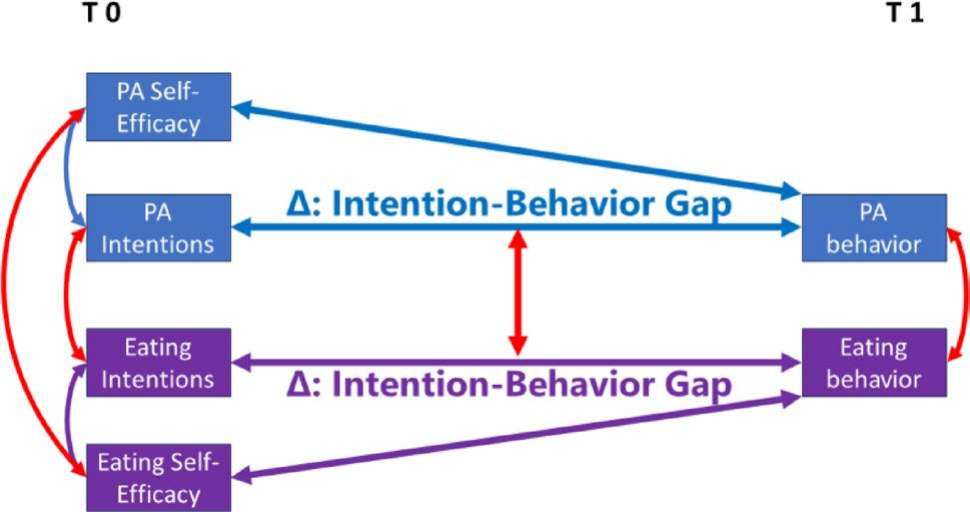
Relation between self-regulatory variables in the assessment schedule. Intention-behavior gaps were calculated as the difference between intentions at T0 (e.g. reported prospectively on Monday night) and behavior at T1 (e.g. reported retrospectively on Tuesday night). Multilevel correlations assessed relations between variables within (blue and purple arrows) and between behavioral domains (red arrows) (Color figure online)

To analyze temporal relations between variables, we created a “lagged” version of the variables intention, self-efficacy, and behavior. In this case, the index T0 indicates the value for a given variable on one day while the index T1 indicates the value for the same variable on the subsequent day (see Fig. 2). To disentangle within- and between-participants effects, predictors were centered within individuals by subtracting the within-subject mean from the individual scores (Van De Pol & Wright, 2009). The within-subject centered variable represents the daily deviation of an individual value from that person’s mean and its effect can therefore be interpreted as day-to-day fluctuations. Missing data were removed upon model fitting.

We calculated a stress score by averaging the two stress items, following earlier studies (Reichenberger et al., 2021; Schultchen et al., 2019). To investigate relations between variables, we computed multilevel correlations between all variables of interest (intention, self-efficacy, and behavior for both behaviors) with random intercepts. To identify temporal relations, we also calculated correlations of variables at T0 and T1. To assess successful intention translation, we then calculated IBGs by subtracting behavior reported at T1 from intentions for that behavior reported at T0. This variable can vary from − 100 (intention value of zero and behavior of 100, indicating that the person did not intend to engage in the behavior but then did) to + 100 (intention of 100 and behavior of zero, indicating that the person intended to engage in the behavior a lot but then did not engage at all). We then used a multilevel model with random intercepts for participants^2^ to predict the healthy eating IBG with the IBG in PA (with one model) and vice versa (in a second model), always controlling for the person-level mean of the predictor variable. In a final step, we added the variables tiredness, hours of sleep, mood, and stress and their interaction with the respective IBG as predictors. All models further included the within-subject means of all variables as control variables 3. All analyses were conducted in *R* software (R Core Team, 2017) using the packages *correlation* (Makowski et al., 2020), *lmertest* (Kuznetsova et al., 2017), *emmeans* (Lenth, 2023), and *ggplot2* (Wickham, 2016).

## Results

In total, included participants answered 1051 of 1406 (75% prompt compliance, range 28% − 100%) of sent prompts. Table 1 (see above) presents means and standard deviations for all EMA variables. It is obvious that intentions and self-efficacy for both behaviors were generally well above the scale’s midpoint of 50, indicating that participants were eager and confident to engage in both behaviors the next day. Still – and consistent with our previous research (Reichenberger et al., 2019) – values for intentions were higher than for behavior in 59% (PA) and 66% (eating) of days with the average IBG being 9.64 (*sd* = 26.8, i.e., participant’s behavior was falling short of their intentions by 9.64 points) for PA and 10.15 (*sd* = 20.7) for healthy eating.

**Fig. 3:**
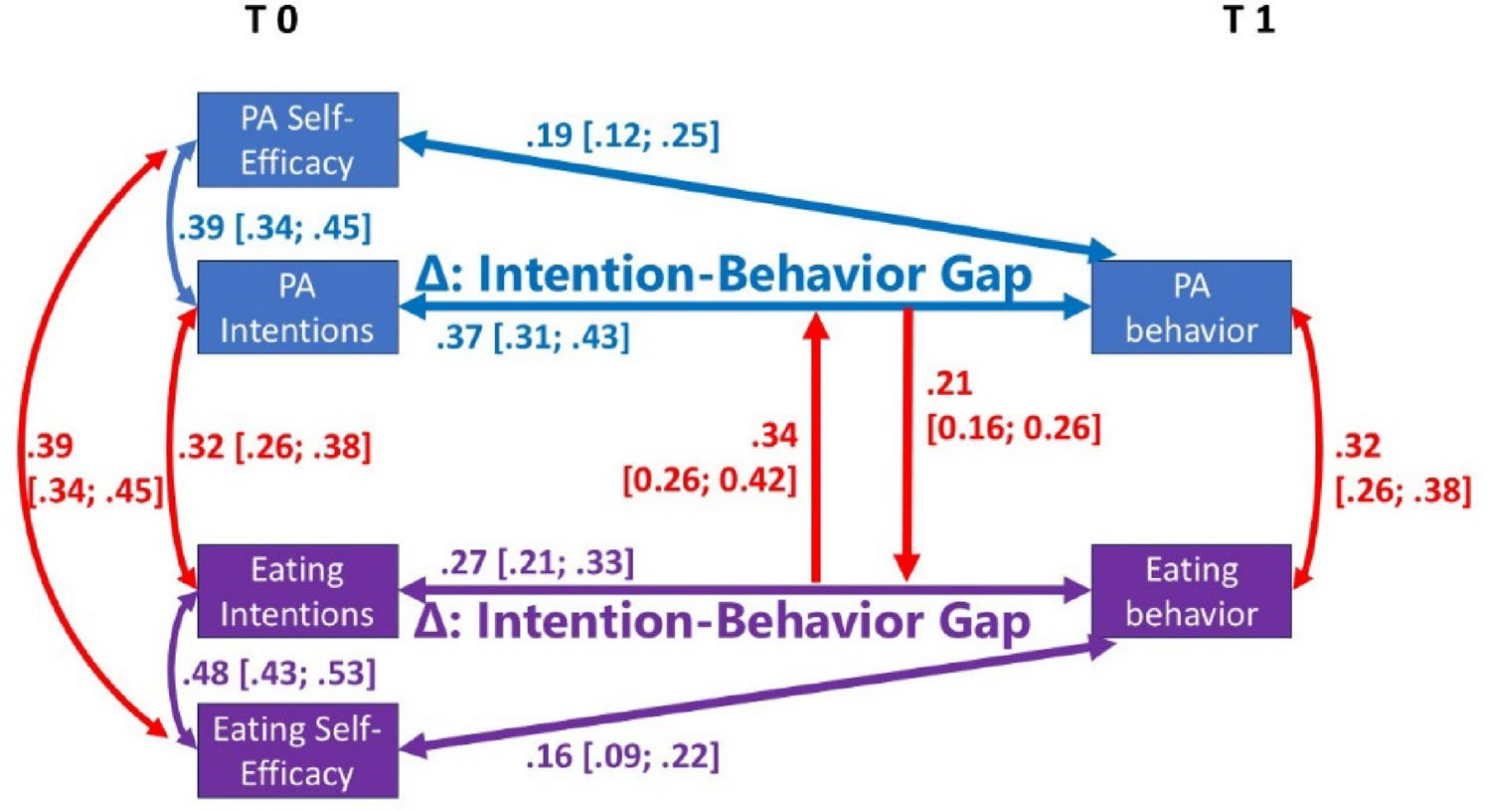
Overview of correlational results. Numbers indicate multilevel correlation coefficients for intentions, behavior, and self-efficacy. For the analyses regarding the intention-behavior gaps, the numbers indicate multilevel model coefficients, all ps < 0.001. Values in square brackets indicate 95% confidence intervals (Color figure online)

In the within-behavior correlation analyses (blue and purple arrows, respectively, in Fig. 3; see the Supplementary Materials for a correlation matrix), intentions and self-efficacy on the same day correlate rather strongly within behavior (0.39, 95% CI = [0.34; 0.45] for PA, 0.48, [0.43; 0.53] for healthy eating). Intentions at T0 and behavior relating to the same day (i.e. reported retrospectively at T1) correlated with 0.37, [0.31; 0.43] for PA and 0.27, [0.21; 0.33] for healthy eating. Regarding cross-behavior correlations (red lines in Fig. 3), intentions, self-efficacy, and behavior for healthy eating correlated with their respective counterparts related to PA at medium strength (between 0.39 and 0.32).

**Fig. 4:**
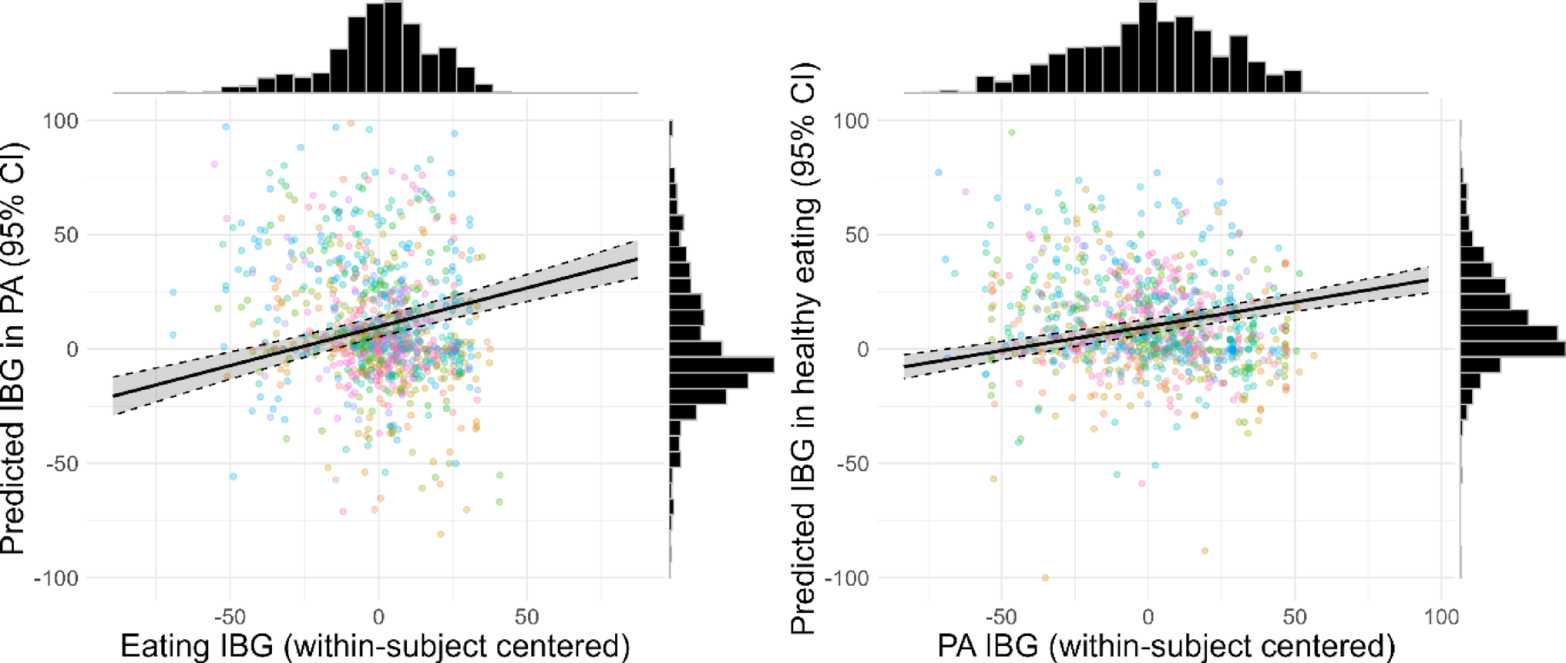
Relation between the intention-behavior gap (IBG) for healthy eating and for PA. The color of the points indicates different participants. Margins display histograms of both variables (Color figure online)

In the cross-behavior model for IBGs (in red color in Fig. 3), the healthy eating IBG significantly predicted the PA IBG (β = 0.34, [0.26; 0.42]; *p* < .001, see Fig. 4, left panel) and vice versa (β = 0.21, [0.16; 0.26]; *p* < .001, Fig. 4, right panel). For the models including potential moderators, we found that better mood was associated with smaller IBGs in both PA (β = − 0.21, [− 0.33; − 0.09]; *p* = .001) and healthy eating (β = − 0.16, [− 0.26; − 0.08]; *p* = .001) but none of the variables significantly moderated the cross-behavior relations (*p*s > 0.22). Importantly, the cross-behavior relations remained highly significant (both *p*s < 0.001) in the presence of these potential moderators.

## Discussion

This exploratory study assessed the day-to-day self-regulation process for healthy eating behavior and physical activity in participants of a health promotion course. Results showed positive day-level cross-behavior relations between intentions, self-efficacy, and self-reported behavior in participants with a higher-order health goal that requires engaging in both behaviors. Intention implementation for eating behavior and physical activity also positively covaried on a day-level, that is, days with a smaller IBG in healthy eating were also days with a smaller IBG in PA and this relation was independent of other factors supposedly influencing self-regulation (stress, mood, sleep, and tiredness).

We could confirm relationships between variables *within* behavioral domains as hypothesized by common models of self-regulation: higher intentions are associated with higher self-efficacy and with more behavior. Crucially, while classic models are based on *between-subjects* data this study adds to a growing body of research that confirms these relationships on a *within-individual* level (Aulbach et al., 2024; Haag et al., 2023; Inauen et al., 2016; Maher et al., 2016; Pickering et al., 2016; Reichenberger et al., 2019): days with stronger intentions are days with more goal-congruent behavior. That being said, we also found that, on average, behavior fell short of intentions in both behaviors, as evidenced by an above-zero average IBG, in line with earlier findings (Aulbach et al., 2024; Reichenberger et al., 2019; Sheeran, 2002; Sheeran & Webb, 2016).

Crucially, the present data go beyond many earlier studies, as they include two key health behaviors, PA and eating, and related self-regulatory cognitions. We demonstrated that PA and eating behavior were positively associated on three key self-regulatory variables: when participants intended to be more active, they also tended to intend to eat more healthily; when they believed in their ability to move more, they also believed in their ability to eat more healthily; and days with more activity were also characterized by healthier food intake. This positive cross-behavior relation was also present regarding successful intention implementation as operationalized by IBGs: the better intentions for healthy eating were translated into behavior, the better intentions for PA were implemented. This positive relationship was independent of third (day level) variables that should influence self-regulation success (mood, stress, sleep, and feeling tired). This contradicts the resource depletion model of self-control (Baumeister, 2000) which has been increasingly questioned in recent years (Hagger et al., 2016) and our results similarly render a simple ‘zero-sum account’ of PA and eating efforts inaccurate. Instead, some ‘cross energizing’ between eating and PA seems to happen, for example individuals not wanting to ‘spoil’ their successes in one domain by relaxing standards in the other domain. Such non-additive effects in the domain of willpower also seem to be subject to interindividual differences: some individuals’ implicit willpower theories regard willpower as a strictly limited resource while others regard it as non-limited and rather feel that engaging in self-control facilitates further acts of self-control (Bernecker & Job, 2015; Job et al., 2010).

Our results also speak against cybernetic models of self-regulation which would predict that progress towards a health goal through one behavior would lead to “coasting”, that is, reduced effort, in another behavior towards the same goal (Carver & Scheier, 2012; Thürmer et al., 2020). It is important to point out, however, that this only applies when both behaviors are perceived to serve the same higher-order goal. We did not assess this perception but given the target population, framing, and contents of the intervention, it seems reasonable to assume that participants thought of daily PA and healthy eating as serving the same higher-order health goal. However, since individuals engage in eating behaviors (Renner et al., 2012) and PA (Molanorouzi et al., 2015) for a wide range of reasons (and therefore serving different goals), the relation between both behaviors might well differ in other populations and settings.

In this sample, results seem more in line with transfer accounts of self-regulation (Fleig et al., 2014): engaging in one health behavior “energizes” the other behavior. Pathways for such spillover could be positive emotions associated with PA that energize efforts in the eating domain (Schultchen et al., 2019), increased self-efficacy through mastery experience in one behavior (Bandura, 2013; Lippke, 2014), or the transfer of self-regulation techniques and strategies between behaviors (Lippke, 2014). Another possible reason for the positive covariation could be that participants spent successful days with others who self-regulated successfully or functioned as social support for participants’ own regulation in other ways (Fitzsimons & Finkel, 2010; vanDellen et al., 2015). Such a social pathway might be particularly relevant in group settings as in this study.

Thinking about possible implications of our findings for intervention development, providing feedback to participants about the positive PA-eating association might be a fruitful intervention avenue to promote beliefs in transfer effects which, in turn, may lead to healthier behavior patterns (Fleig et al., 2014, 2015). Similarly, this kind of feedback could promote beliefs in willpower being infinite rather than being a limited resource. Such beliefs are associated with more successful self-regulation (Job et al., 2010) and developing them might therefore support health behavior change. In a similar vein, interventions should support this positive association by showing ways how self-regulatory strategies like planning can be implemented in both behavioral domains (Paech & Lippke, 2017). The within-individual EMA based data structure would allow for person adaptive, real-time interventions, also termed just in time adaptive interventions (JITAI, Nahum-Shani et al., 2018).

### Strengths and limitations

One major strength of this study is the recruitment of a sample from a diverse, highly relevant population: participants were in the process of changing at least two relevant health behaviors (PA and eating) and their motivation for doing so is based on their own assessment of potential future and/or already occurring health problems. Collecting data in these populations is important, as a large share of health-behavior related EMA studies are conducted in student samples (Perski et al., 2022) who tend to be young, in the normal weight range, and healthy and therefore might differ substantially regarding their motivation for behavior change. Crucially, they also tend to be more used to regular smartphone use and more interested in using m-health applications than older participants (Nunes et al., 2019). Our results show that the vast majority of course participants were willing to participate in the study (28 out of 34) without compensation. As another strength, data were collected in real life via EMA and are thus temporally close to the behaviors and self-regulatory processes involved and allow examining day-to-day relations between variables. The long period of data collection (eight weeks) is another strength that provides good statistical power on a within-subjects level and allows studying the self-regulation process more long-term than many similar studies (Perski et al., 2022). Finally, the simultaneous assessment of two crucial health behaviors alongside self-regulatory cognitions is rare in the literature and thus offers novel insights.

One limitation is the rather small sample size determined by practical constraints (rather than formal power analysis) that limits statistical power on a between-subject level as well as the predominantly female sample. Aiming to keep participant burden low, we also assessed only a narrow range of potentially relevant variables. These two points combined limit the complexity of possible analyses: for example, examining the role of willpower beliefs (Job et al., 2010) would require data from more participants but would allow examining whether participants who believe willpower to be endless show more positive PA-eating associations than those who believe in limited willpower. Equivalent considerations apply to the role of person-level compensatory vs. transfer cognitions. We further recommend assessing the presence, importance, and exact content of the higher-order health goal which was simply assumed from the context of the current study. Similarly, identifying time-variant variables that helped or hindered translating intentions into behavior would require the assessment of more variables, including those of social and material contexts that can help or hinder self-control (Fitzsimons & Finkel, 2010; Hofmann et al., 2012). Finally, we assessed only perceptions of both behaviors rather than more objective markers. Some studies indicate that perceptions of changes in diet (Szymczak et al., 2021) and PA (Szymczak et al., 2020) can be rather accurate. However, since some nutrients (Gibson, 2006) and different levels of physical activity (Chan et al., 2019) might have physiological and psychological effects relevant to self-regulation, those should be assessed as objectively as possible in future studies. Finally, interpretation of results is tied to our specific sample who were actively participating in a health promotion course. Future studies should aim to replicate these findings with a study design that includes a control condition where participants do not receive an active intervention. Further conducting similar studies in participants who have no specific higher-order health goals will elucidate to what degree the current findings hold for the general population.

### Conclusion

This exploratory study demonstrated positive day-level cross-behavior associations between behavior, self-regulatory cognitions, and successful and failed intention implementation between PA and healthy eating in participants of a health promotion course. Crucially, results show that PA and healthy eating positively covary on a day-level on all examined variables, speaking against simple cybernetic and limited resource accounts of self-regulation and are more in line with transfer effects derived from social cognitive models in individuals who strive for a higher-order health goal. These results underline the suggestion that understanding one health behavior (healthy eating) requires knowledge of other health behaviors (physical activity). Future research should identify variables on the within- and between-subject level that could further explain this association and identify which processes might facilitate positive transfer effects from one behavior to the other.

## Data Availability

Data, analysis code, Supplementary Material, and the pre-print of this manuscript can be found at https://osf.io/ubvn5/.

## Footnotes

1 As this threshold is arbitrary, we also conducted all analyses with the full dataset which did not alter the general pattern of results.

2 We first estimated models with random slopes which led to convergence issues. Therefore, we simplified the model to include only random intercepts.

3 We also checked the influence of the progression of time by adding the day within the study as a predictor. The effect of time was not significant and was thus omitted from all models.
